# Turn up the cellular power generator with vitamin E analogue formulation†
†Electronic supplementary information (ESI) available: Details of materials, instrumentation, methods, and additional figures. See DOI: 10.1039/c6sc00481d


**DOI:** 10.1039/c6sc00481d

**Published:** 2016-05-09

**Authors:** Ru Wen, Shanta Dhar

**Affiliations:** a NanoTherapeutics Research Laboratory , Department of Chemistry , University of Georgia , Room 679 , Athens , GA 30602 , USA . Email: shanta@uga.edu ; Fax: +1-706-542-9454 ; Tel: +1-706-542-1012 ; http://shanta.uga.edu/

## Abstract

The down regulation of the cellular power generator, adenosine triphosphate (ATP) synthase, in various cancer cells plays an obstructive role in mitochondria-mediated cell death. Cancer cells up-regulate ATPase inhibitory factor 1 (IF1) and down-regulate β-F_1_-ATPase of ATP synthase to enhance aerobic glycolysis for tumor growth *via* inhibiting total ATP synthase activity in the oxidative phosphorylation (OXPHOS) pathway. Alpha-tocopheryl succinate (α-TOS), one of the most bioactive derivatives of vitamin E, can selectively induce apoptosis in numerous cancer cells. The cancer cell selective apoptosis inducing property of α-TOS is correlated to: mitochondrial destabilization, inhibition of anti-apoptotic B cell lymphoma 2 (Bcl2) and protein kinase C (PKC), caspase 3 activation, production of mitochondrial reactive oxygen species (ROS), and inhibition of succinate dehydrogenase activity of mitochondrial complex II, and interaction with complex I to some extent. There is no report which elucidates the effects of α-TOS on the cellular power generator, complex V or ATP synthase. Here, we report the activation of mitochondrial ATP synthase using a suitably designed chemical formulation of α-TOS for the first time. A mitochondria targeted α-TOS nanoparticle formulation demonstrated enhanced cytotoxicity and mitochondrial activities in cancer cells by inhibiting Bcl2 protein and activating ATP synthase. The modulation of ATP synthase in cancer cells by the engineered formulation of α-TOS can be promising for solid cancers with compromised ATP synthase.

## Abstract 2

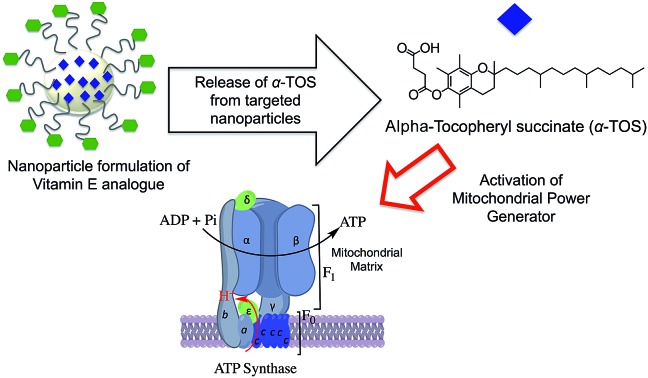
We report cancer cell selective activation of mitochondrial ATP synthase using a suitably designed chemical formulation of alpha-tocopheryl succinate.

## Introduction

**Scheme 1:**
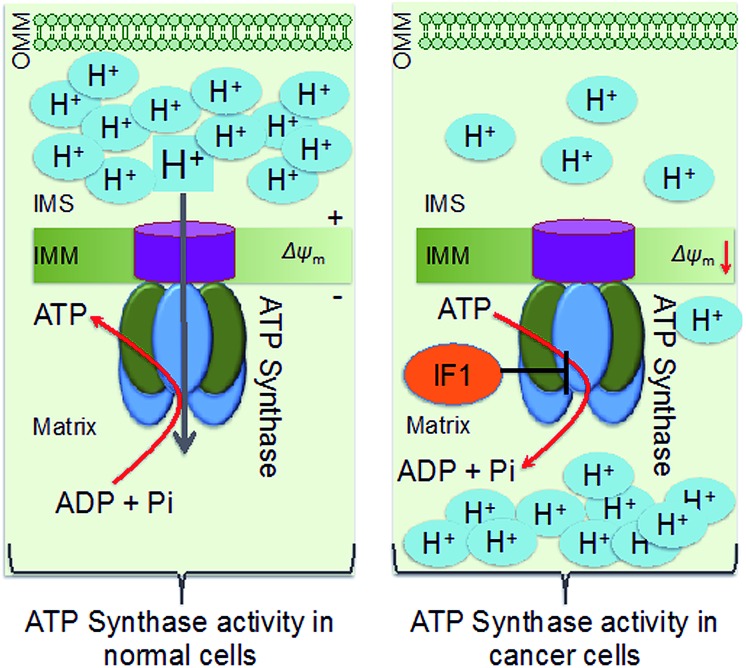
Schematic representation of differences in ATP synthase in normal and cancer cell mitochondria. OMM: outer mitochondrial membrane; IMS: intermembrane space; IMM: inner mitochondrial membrane.

Down-regulation of the cellular power generator, ATP synthase, is a signature bioenergetic phenomenon of several tumors, such as cancers of the liver, kidney, colon, lung, and breast.^1^–^4^ Dysfunctional mitochondrial bioenergetics related to the down regulation of ATP synthase in cancer cells limits electron flux in the respiratory chain due to coupling between mitochondrial respiration and oxidative phosphorylation (OXPHOS), and results in resistance towards mitochondria-mediated cancer cell death. Mitochondrial ATP synthase, or F_1_F_0_-ATP synthase, is a unique complex of 16 subunits of α3β3γδ and ε, that form the F_1_ component; a, b, c, d, e, f, g, A6L, oligomycin sensitivity-conferring protein (OSCP), and coupling factor 6 that form the F_0_ component; the stator, and an intrinsic inhibitor protein, ATPase inhibitory factor 1 (IF1). The decreased activity of ATP synthase in cancer cells arises from a combination of up-regulated expression of IF1 and down-regulation of β-F_1_-ATPase, resulting in enhanced aerobic glycolysis (Scheme 1).^5^ ATP synthase activity and protein expression in colon cancer cells were reported to be down regulated under 5-fluorouracil (5-FU) resistance.^4^ The suppression of ATP synthase subunits is dependent on cancer cell lines. Clinical studies demonstrated that patients possessed a longer life expectancy when there is a higher tumor expression level of β-F_1_-ATPase.^6^ Thus, ATP synthase activity enhancement in cancer cells can modulate the mitochondrial bioenergetic functions to cause cancer cell death in unique ways. However, there are not many therapeutic agents that can enhance the activity of ATP synthase in a therapeutically beneficial way. Defects in ATP synthase are involved in difficult to treat tumors. Given that this ATP generator is located in the inner membrane of mitochondria and in a highly concentrated manner in inner membrane cristae as a part of the ATP synthasome and in association with inorganic phosphate carriers and the adenine nucleotide translocase,^4^ it is extremely difficult to access ATP synthase for therapeutic modulation.

**Fig. 1:**
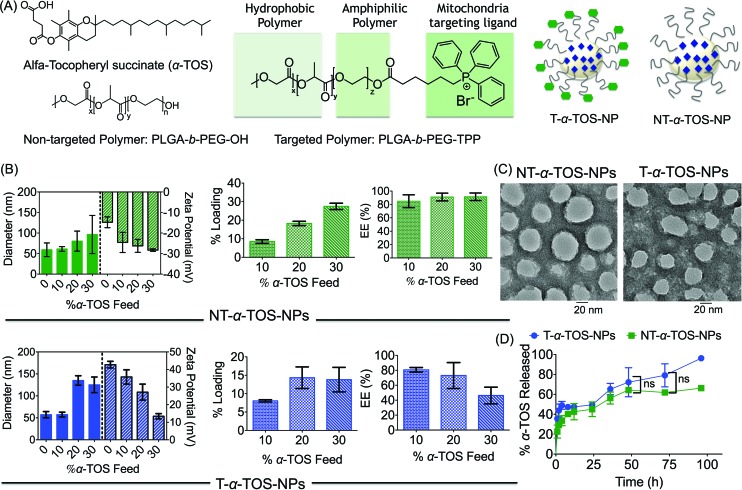
(A) Structures of targeted, non-targeted polymers, α-TOS, and schematic representations of targeted-α-TOS-NPs (T-α-TOS-NPs) and non-targeted-α-TOS-NPs (NT-α-TOS-NPs). (B) Comparison of diameter, zeta potential, percent loading and EE of library of T and NT NPs prepared with varied α-TOS feeds. (C) TEM of T/NP-α-TOS-NPs prepared with 10% α-TOS feed. (D) Release kinetics of α-TOS from T and NT NPs under physiological conditions of temperature 37 °C, pH 7.4. ns: non significant.

Alpha-tocopheryl succinate (α-TOS) is the most bioactive vitamin E analogue, among α-tocopherol, α-tocopheryl acetate, and α-tocopheryl nicotinate, that participates in selective apoptosis induction in cancer cells.^7^ α-TOS selectively inhibits proliferation and induces apoptosis in various cancer cells, such as leukemia, breast, colorectal, and prostate cancer cells, over normal cells.^8^ The pro-apoptotic and anti-neoplastic properties of α-TOS are related to mitochondrial destabilization, accessing several targets located in this complex organelle.^9^ A brief list of biological actions and targets of α-TOS includes: mitochondrial destabilization, inhibition of anti-apoptotic B cell lymphoma 2 (Bcl2),^10^ protein kinase C (PKC), and mitochondrial complex II, caspase 3 activation, generation of mitochondrial reactive oxygen species (ROS),^11^ and inhibition of mitochondrial complex I to some extent.^12^–^14^ A mitochondria targeted α-TOS formulation, MitoVES, was developed by conjugating α-TOS with a mitochondria-targeting triphenylphosphonium (TPP) cation.^15^,^16^ However, no work thus far has looked into the activity of α-TOS on mitochondrial complex V or ATP synthase. In this work, we aimed to investigate the effects of this vitamin E analogue on ATP synthase by developing a chemical formulation with abilities to associate α-TOS with the mitochondrial space. We developed a chemical formulation of α-TOS to access ATP synthase in cancer cells for therapeutic benefits. Here, we report the unexplored activity of α-TOS when it is delivered to the mitochondria using a biodegradable controlled release polymeric nanoparticle (NP) (Fig. 1A). The mitochondria targeted α-TOS-NPs demonstrated the activation of ATP synthase. Given that the down-expression of the β subunit of ATP synthase is associated with the mitochondrial bioenergetics dysfunctions of various cancers, such as cancers of the liver, kidney, colon, lung, and breast,^2^–^4^,^17^ we believe that the ATP synthase activation property of the mitochondria targeted NP formulation of α-TOS can be important when explored further under *in vivo* settings.

## Results and discussion

### Construction of α-TOS NPs to access ATP synthase

A block copolymer of poly(lactic-*co*-glycolic acid) (PLGA)–polyethylene glycol (PEG) (PLGA-*b*-PEG-OH) and mitochondria targeting TPP cation, which utilizes the mitochondrial membrane potential (Δ*ψ*_m_), were used to construct a mitochondria targeted polymer PLGA-*b*-PEG-TPP.^18^–^22^ Mitochondria-targeted α-TOS encapsulated T-α-TOS-NPs and the control non-targeted NT-α-TOS-NPs were constructed using PLGA-*b*-PEG-TPP and PLGA-*b*-PEG-OH polymers, respectively (Fig. 1A). A library of targeted and non-targeted NPs were prepared using varied percent feeds of α-TOS with respect to PLGA-*b*-PEG-TPP or PLGA-*b*-PEG-OH, for NP property optimization in terms of their diameter, charge, and payload loading (Fig. 1B). Determination of the hydrodynamic diameter (*Z*_average_), zeta potential, and polydispersity index (PDI) of the α-TOS loaded NPs by dynamic light scattering (DLS) indicated that a higher α-TOS feed results in larger particles. The increase in size with the increased feed of α-TOS was particularly more significant for the T-α-TOS-NPs (Fig. 1B, ESI Fig. S1†). The T-α-TOS-NPs demonstrated a decreasing positive surface charge as the α-TOS feed was increased. The NT-α-TOS-NPs showed less negatively charged surfaces with increasing α-TOS feed (Fig. 1B, ESI Fig. S1†). The targeted NPs prepared from 10% α-TOS feed, with a diameter of 57.4 ± 5.5 nm and a zeta potential of +35.8 ± 4.1 mV and the non-targeted NPs with a diameter of 61.2 ± 5.7 nm and a zeta potential of –24.6 ± 4.9 mV were found to be most suitable for our purpose (Fig. 1B). High performance liquid chromatography (HPLC) analysis indicated that the loading of α-TOS in the targeted or non-targeted NPs was ∼8% with an encapsulation efficiency (EE) of 80% for the 10% α-TOS feed (Fig. 1B, ESI Fig. S2†). In our studies, NPs with a 10% feed of α-TOS with respect to the polymer were used. A homogeneous spherical population of α-TOS-NPs was confirmed by transmission electron microscopy (TEM) analysis (Fig. 1C). Investigation of α-TOS release from T and NT NPs under physiological conditions of pH 7.4 at 37 °C indicated that both targeted and non-targeted NPs were able to release α-TOS in a well-controlled manner, and there was no significant difference in the extent of α-TOS release between the targeted and non-targeted NPs (Fig. 1D).

### Unique activities of α-TOS NPs in cancer cells

**Fig. 2:**
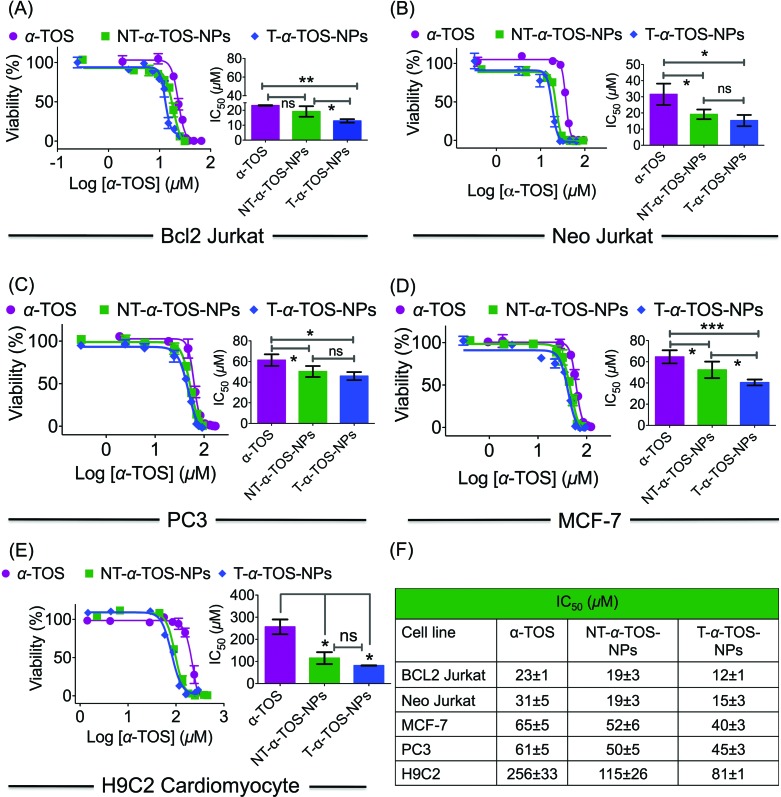
(A–D) Cellular toxicity of α-TOS, T-α-TOS-NPs, and NT-α-TOS-NPs in different cancer cell lines. (E) Relatively lower cytotoxic effects of α-TOS, T-α-TOS-NPs, and NT-α-TOS-NPs in normal H9C2 cardiomyocytes. (F) A comparison of toxicity of α-TOS, T-α-TOS-NPs, NT-α-TOS-NPs in cancer and normal cells. ***P < 0.001; **P = 0.001–0.01; *P < 0.05.

The cytotoxic activities of NT/T-α-TOS-NPs and α-TOS were evaluated in Bcl2 expressing Bcl2 Jurkat and knock out Neo Jurkat cells. The Bcl2 levels in these two cell types were evaluated by western blot. In both the cancer cell lines, the T-α-TOS-NPs demonstrated significantly higher cytotoxic effects than free α-TOS (Fig. 2A and B). In Bcl2 overexpressing Jurkat cells, when α-TOS was delivered with mitochondria targeted T-α-TOS-NPs, its activity was significantly increased compared to that of non-targeted NT-α-TOS-NPs; however, their activities were not statistically different in Bcl2 negative Neo Jurkat cells (Fig. 2). This indicated that in Bcl2 overexpressing cells, delivering α-TOS to the mitochondria is beneficial since the target Bcl2 is located at the OMM. In breast cancer MCF-7 and prostate cancer PC-3 cell lines, the activity of T-α-TOS-NPs was found to be better than free α-TOS or NT-α-TOS-NPs (Fig. 2). It was particularly interesting to observe that T/NT-α-TOS-NPs were less toxic in normal heart cells; these experiments were carried out on rat cardiomyocyte H9C2 cells (Fig. 2).

**Fig. 3:**
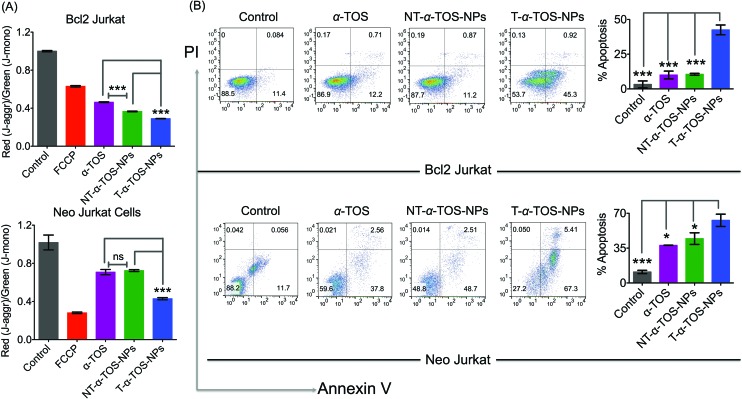
(A) Changes in mitochondrial membrane potential in Bcl2 Jurkat and Neo Jurkat cells in the presence of α-TOS, T-α-TOS-NPs, and NT-α-TOS-NPs. (B) Comparison of extent of apoptosis in Bcl2 Jurkat and Neo Jurkat cells in the presence of α-TOS, T-α-TOS-NPs, NT-α-TOS-NPs by Annexin V-PI assay. ns: non significant. ***P < 0.001; **P = 0.001–0.01; *P < 0.05.

Cell death pathways were determined by analyzing the state of Δ*ψ*_m_ of Bcl2 and Neo Jurkat cells upon treatment with α-TOS or T/NT-α-TOS-NPs (Fig. 3A, ESI Fig. S3†). As a control, carbonyl cyanide 4-(trifluoromethoxy)phenylhydrazone (FCCP), which is an uncoupler of the mitochondrial membrane, was used. These studies indicated that the collapse of Δ*ψ*_m_ is more significant in T-α-TOS NP treated cells compared to α-TOS or NT-α-TOS-NP treated cells. Cellular apoptosis was analyzed by Annexin V/propidium iodide (PI) staining after treatment with α-TOS or T/NT-α-TOS-NPs in Bcl2 and Neo Jurkat cells. These studies indicated increased cell apoptosis by T-α-TOS-NPs compared to α-TOS or NT-α-TOS-NPs in both the cell lines; however, the increased apoptotic properties of T-α-TOS-NPs were more drastic in Bcl2 Jurkat cells (Fig. 3B).

### Inhibition of Bcl2 and induction of apoptosis inducing factor

**Fig. 4:**
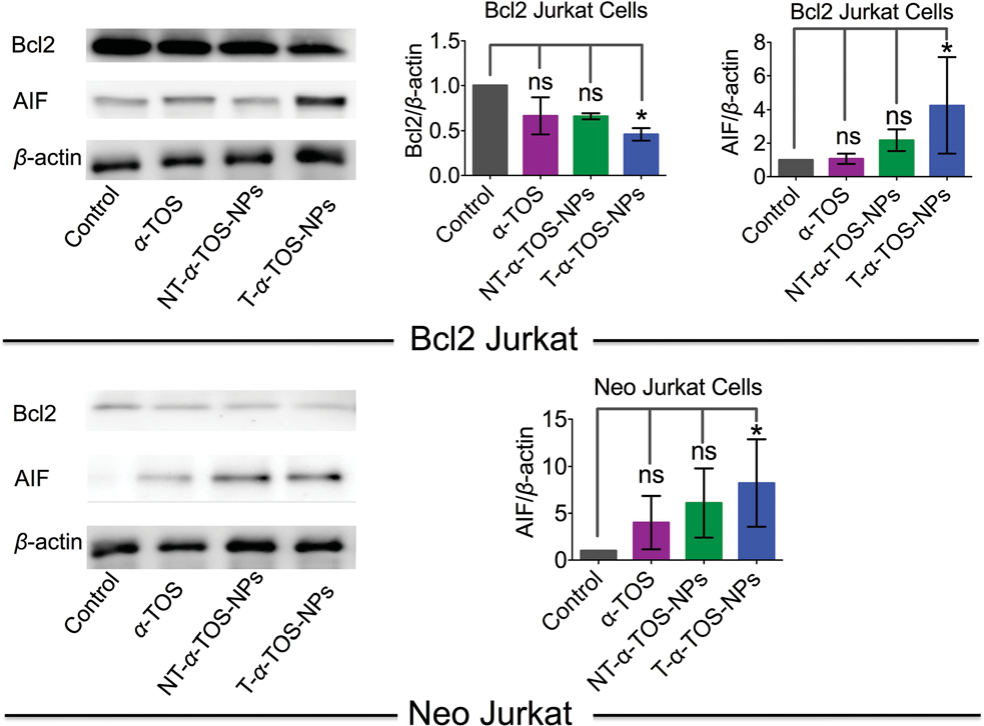
Representative western blot images and quantification of Bcl2 and AIF proteins in Bcl2 Jurkat and Neo Jurkat cells treated with α-TOS and its T/NT NPs.

Bcl2, a mitochondrial membrane protein mainly located in the OMM, prevents most types of cell apoptosis.^23^,^24^ α-TOS induces apoptosis by regulating the Bcl-xL/Bcl2 pathways, in which α-TOS disrupts the association of Bak Bcl2 homology 3 (BH3) peptide to Bcl-xL and Bcl2, resulting in caspase-dependent apoptosis.^10^,^11^ Apoptosis-inducing factor (AIF) is a mitochondrial intermembrane flavoprotein that induces caspase-independent apoptotic cascade in the presence of death stimuli.^25^ Western blot analyses were carried out in Bcl2 and Neo Jurkat cells to investigate whether the T/NT-α-TOS-NPs treatment modulated the expression of these key signal proteins (Fig. 4). We observed ∼50% down expression of the Bcl2 protein in Bcl2 Jurkat cells when treated with T-α-TOS-NPs. Inhibition of Bcl2 in α-TOS or NT-α-TOS-NP treated Bcl2 Jurkat cells was much less compared to that of targeted NPs (Fig. 4). For T-α-TOS-NPs, AIF was up regulated both in Bcl2 Jurkat and in Neo Jurkat cells (Fig. 4). These results indicate that T-α-TOS-NPs are able to modulate the mitochondrial proteins Bcl2 and AIF for cellular apoptosis.

### Effects of T-α-TOS-NP on mitochondrial bioenergetics and respiratory complexes

**Fig. 5:**
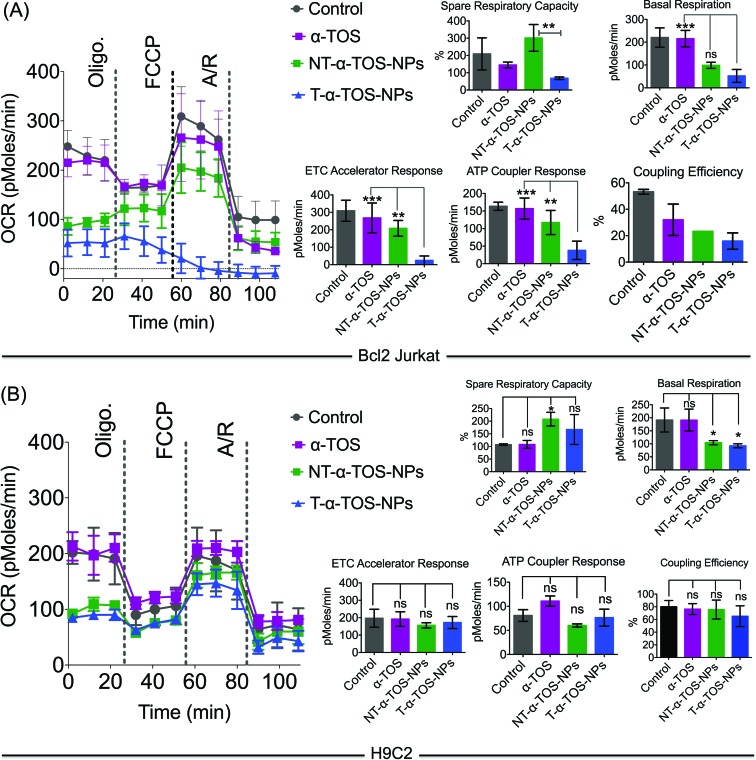
Mitochondrial activity of α-TOS, T-α-TOS-NPs, and NT-α-TOS-NPs in (A) Bcl2 Jurkat and (B) H9C2 cells. From these studies, it was found that T-α-TOS-NPs cause the stimulation of ATP synthase upon injection of oligomycin only in cancer cells, and not in normal cells. ns: non significant. ***P < 0.001; **P = 0.001–0.01. A: antimycin A; R: rotenone.

The effects of T/NT-α-TOS-NPs and α-TOS on the bioenergetics of Bcl2 Jurkat cells were evaluated by performing a mito stress assay using an XF24 extracellular flux analyzer (Fig. 5). Free α-TOS treated cells showed similar trends under the influence of the ATP synthase inhibitor oligomycin, FCCP, which is an uncoupling agent, and a mixture of antimycin A (A), a mitochondrial complex III inhibitor and rotenone (R), a mitochondrial complex I inhibitor as seen with the control cells, demonstrating no significant activity on mitochondrial energetics and respiration at the concentration used (Fig. 5).

At the same α-TOS concentration, after oligomycin injection, the oxygen consumption rates (OCRs) in the presence of T-α-TOS-NPs and NT-α-TOS-NPs did not experience reduction compared to OCR prior to oligomycin addition, which one would predict is due to ATP synthase inhibition (Fig. 5). This suggested that when delivered with a NP, α-TOS is either participating in ATP synthase activation or prohibiting oligomycin to inhibit ATP synthase. When FCCP was injected, an increase in OCR was observed for the NT-α-TOS-NP treated cells; however, the OCR levels in T-α-TOS-NP treated cells declined, indicating that Δ*ψ*_m_ collapsed for this treatment. Compared to free α-TOS, the NT-α-TOS-NP treatment had more effects on the mitochondrial respiration of these cells. The injection of a mixture of A and R resulted in the shut down of mitochondrial respiration for every group (Fig. 5A).

In normal H9C2 cells, α-TOS, NT/T-α-TOS-NPs showed similar trends to the control when mitochondrial stress inducers, oligomycin, FCCP, and A/R were injected (Fig. 5B). In particular, unlike cancer cells, the NT/T-α-TOS-NPs treated H9C2 cells did not experience an increase in OCR when oligomycin was injected. This indicated that the activity of NT/T-α-TOS-NPs on ATP synthase is cancer cell specific.

### T-α-TOS-NP-induced activation of the power generator, ATP synthase

**Fig. 6:**
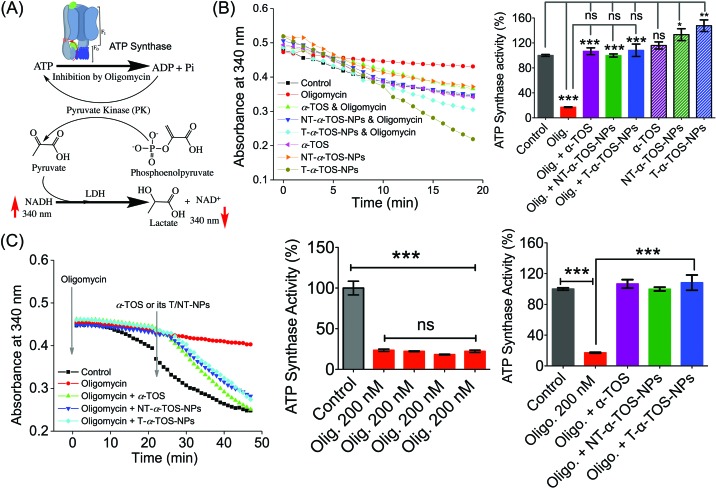
(A) Schematic representation of ATP synthase activity used in our experiment. (B) Stimulation of ATP synthase activity by α-TOS, T-α-TOS-NPs, and NT-α-TOS-NPs, in the presence or absence of the ATP synthase inhibitor oligomycin. (C) Recovery of oligomycin inhibited ATP synthase activity by α-TOS or T/NT-α-TOS-NPs. ns: non significant. ***P < 0.001; **P = 0.001–0.01.

**Fig. 7:**
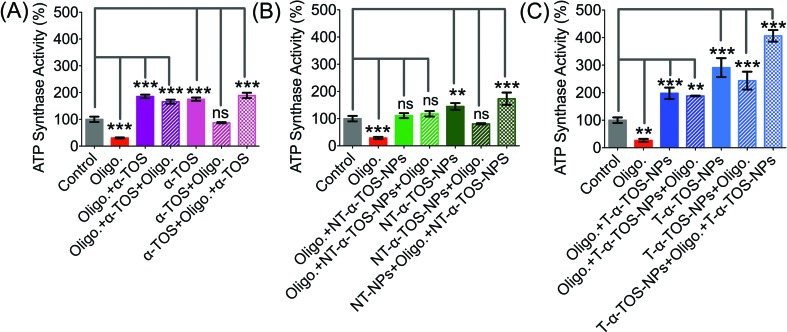
Recycled ATP synthase activation by α-TOS (A: Left), NT-α-TOS-NPs (B: Middle), and T-α-TOS-NPs (C: Right) in the presence of oligomycin. ns: non significant. ***P < 0.001; **P = 0.001–0.01.

The observation that T/NT-α-TOS-NPs promote ATP synthase activity either by enhancing its action or by inhibiting oligomycin prompted us to investigate the effects of these NPs on ATP synthase. Detailed ATP synthase activity assays were carried out to shed light on this observation. ATP synthase activity was determined using an *in vitro* assay on isolated bovine heart mitochondria in the presence of substrates for ATP synthesis. Mitochondrial ATP synthase activity was monitored by following ATP hydrolysis.^26^–^28^ ATP synthase was exposed to the substrate buffer by solubilizing isolated bovine heart mitochondria. The regeneration of adenosine diphosphate (ADP) coupled with the oxidative reaction of NADH to NAD^+^ in the presence of the pyruvate kinase (PK) and lactate dehydrogenase (LDH) was used in this experiment (Fig. 6A). ATP synthase activity enhancement was found to be highest when T-α-TOS-NPs were used (Fig. 6B). There was no significant difference between free α-TOS and the control. When oligomycin was used as an inhibitor of ATP synthase, it was possible to protect the activity of this enzyme in the presence of α-TOS or T/NT-α-TOS-NPs (Fig. 6C). Any possibility of complex formation between oligomycin and α-TOS was ruled out by performing HPLC analyses (ESI Fig. S4†). This study demonstrated that α-TOS does not interact with oligomycin. Thus, α-TOS recovers the ATP synthase activity in a different pathway, rather than just reacting with oligomycin. It was possible to recover the oligomycin inhibited ATP synthase activity by either free α-TOS or T/NT-α-TOS-NPs (Fig. 6C). We further investigated whether this ATP synthase activation can be recycled; first by the addition of oligomycin or T-α-TOS-NPs, then T-α-TOS-NPs or oligomycin, and then oligomycin or T-α-TOS-NPs. In this fashion, it was possible to modulate the ATP synthase activities (Fig. 7). It was possible for the T-α-TOS-NPs to recycle ATP synthase activity. ATP synthase recycling activity was also observed with α-TOS and NT-α-TOS-NPs (Fig. 7A and B) in the presence of oligomycin. However, the extent of ATP synthase activation was less for α-TOS or NT-α-TOS-NPs, in contrast to T-α-TOS-NPs. We believe that the enhanced ATP synthase activation by T-α-TOS-NPs is due to close proximity of α-TOS to ATP synthase when delivered with a mitochondria targeted NP.

### ATP and lactate levels in treated cells

**Fig. 8:**
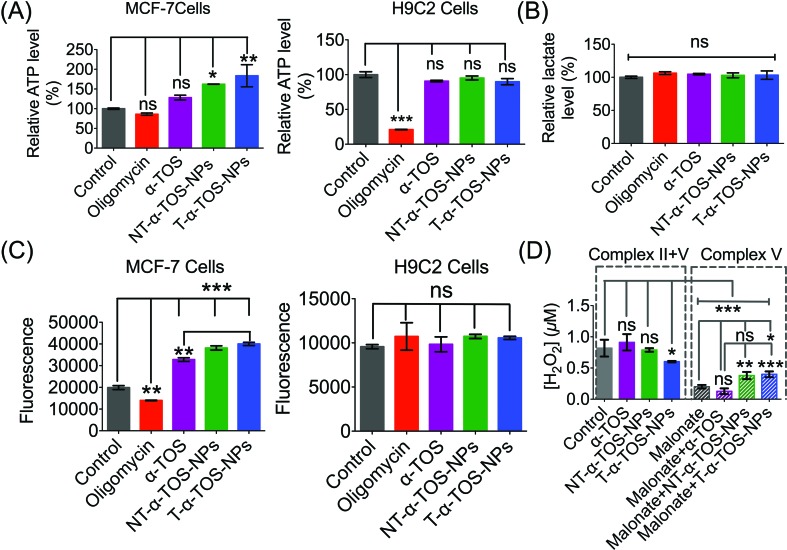
(A) Increased production of ATP in MCF-7 breast cancer cells in the presence of α-TOS, T-α-TOS-NPs, and NT-α-TOS-NPs. There was no significant increase in the ATP level in normal H9C2 cardiomyocytes in the presence of α-TOS, T-α-TOS-NPs, and NT-α-TOS-NPs. (B) Relative lactate level in MCF-7 cells treated with oligomycin, α-TOS, or T/NT-α-TOS-NPs. (C) Increased ROS production in MCF-7 in presence of α-TOS, T-α-TOS-NPs, and NT-α-TOS-NPs. There was no significant increase in the ROS level in normal H9C2 cardiomyocytes in the presence of α-TOS, T-α-TOS-NPs, and NT-α-TOS-NPs. (D) H_2_O_2_ production in MCF-7 cells by an Amplex Red-hydrogen peroxide/peroxidase assay in the presence of α-TOS, NT-α-TOS-NPs, or T-α-TOS-NPs (40 μM with respect to α-TOS) without or with 100 mM malonate for 24 h. ns: non significant. ***P < 0.001; **P = 0.001–0.01.

The enhanced ATP synthase activity was further confirmed by the increased production of ATP in MCF-7 breast cancer cells in the presence of T/NT-α-TOS-NPs; however, free α-TOS did not result in any significant increase in ATP (Fig. 8A). This observation further strengthens the claim that T/NT-α-TOS-NPs participate in ATP synthase activation. Oligomycin at a concentration of 100 μM did not show inhibition of the ATP level in MCF-7 cells. This is most likely due to the Warburg effect that exists in cancer cells undergoing glycolytic ATP generation.^29^ However, there was no significant difference in ATP levels compared to the control when H9C2 normal cells were treated with α-TOS or T/NT-α-TOS-NPs (Fig. 8A). Oligomycin at a concentration of 25 μM significantly decreased the ATP production by H9C2 normal cells (Fig. 8A).

We then examined whether the activated OXPHOS pathway affected glycolysis in α-TOS or T/NT-α-TOS-NPs treated MCF-7 cells. No significant change in the lactate level was observed when α-TOS or T/NT-α-TOS-NP was used. These results indicated that the T/NT-α-TOS-NPs activate ATP synthase activity without affecting the glycolytic pathway (Fig. 8B).

### Cancer cell selective ROS production

The inhibition of mitochondrial respiration chain complexes I and II by α-TOS is well documented.^12^,^14^ Our current study discovered an additional cellular activity of α-TOS when delivered with a NP formulation. This new pathway uses the activation of ATP synthase or complex V. α-TOS is also known to increase Ca^2+^ uptake in the mitochondria.^30^ The activity of ATP synthase can be enhanced by intra-mitochondrial Ca^2+^. This is supported by the fact that when a Ca^2+^ blocker such as ruthenium red is used, ATP synthase activity can be abolished.^31^–^33^ Further, an overload of Ca^2+^ in the mitochondria causes mitochondrial membrane permeability damage and excess ROS production, which eventually leads to apoptotic cell death.^34^,^35^ ATP synthase mediates apoptosis by ROS production, and down-regulation of ATP synthase is an approach used by cancer cells to escape ROS related apoptosis.^36^ The expression of ATP synthase is known to be suppressed in cancer cells which undergo a glycolytic pathway for energy generation.^37^ The up regulated expression of IF1 of ATP synthase is modulated in cancer cells with the Warburg phenotype of enhanced aerobic glycolysis.^5^ It was also reported that down regulation of ATP synthase activity in colon cancer cells is observed under chemo-resistance.^4^ Thus, the activation of ATP synthase provides a strategy to induce cancer cell apoptosis by increasing ROS production.

We measured ROS production by breast cancer MCF-7 cells in response to α-TOS or T/NT-α-TOS-NPs. These studies indicated that ROS was increased significantly compared to the control cells in presence of α-TOS or its NP formulation. This increase in ROS was more significant for T-α-TOS-NP treated cells (Fig. 8C). When the same study was conducted in normal H9C2 cardiomyocytes, no enhancement in ROS was observed with α-TOS or its NPs (Fig. 8C). It is reported that cancer cells are more sensitive to α-TOS and may uptake more α-TOS compared to normal cells.^7^ We believe that the differences in ROS production in cancer and normal cells are due to less uptake of free α-TOS by normal cells, which maintain a more alkaline pH, favouring the deprotonated form of α-TOS, compared to the uptake in cancer cells, which have a characteristically acidic cellular milieu. Furthermore, in regards to T-α-TOS-NPs, less hydrolysis of α-TOS should occur in normal cells than in cancer cells due to the relatively lower abundance of esterases.

Previous studies supported the hypothesis that apoptosis of cancer cells by α-TOS is notably *via* inhibition of complex II. Our findings show a contribution of complex V towards α-TOS activity when using suitably engineered NPs. Thus, we investigated the extent of complex V contribution towards the enhanced activity of α-TOS when used in a NP platform. We studied the contributions of complex II and V by measuring the H_2_O_2_ release profile from MCF-7 cells. Malonate is reported to inhibit complex II activity towards H_2_O_2_ production^38^ and thus we introduced it to inhibit complex II contribution in the presence of T/NT-α-TOS NPs or free α-TOS. Interestingly, we found that the NP formulations of α-TOS (T/NT-α-TOS-NPs) have greater H_2_O_2_ production attributed to complex V activity compared to free α-TOS (Fig. 8D). This indicated that T-α-TOS-NPs are most effective in activating complex V.

## Conclusions

Targeted α-TOS-NP was developed to modulate cancer cell function by accessing targets at the mitochondrial membrane. Engineered NP formulations of α-TOS exhibited enhanced cytotoxicity and mitochondrial activity in cancer cells compared to free α-TOS. Mitochondria-targeted α-TOS-NPs showed the highest mitochondrial activity related to apoptosis, Bcl2/AIF regulation, and ATP synthase. This was likely due to enhanced apoptosis by ROS generation through increased ATP synthase activity. Therefore, T-α-TOS-NP is a promising formulation of vitamin E with abilities to modulate the cellular power generator ATP synthase in cancer cells. T-α-TOS-NP may provide a powerful therapeutic tool against solid cancers with a down-regulated β subunit of ATP synthase. Application of this formulation of vitamin E should be explored further for new therapeutic gains.

## Experimental details

### Materials, instrumentations, and methods

A detailed description of materials, instruments, and methods can be found in the ESI.†

### Statistics

All data were expressed as mean ± S.D (standard deviation). Statistical analyses were performed using GraphPad Prism® software v. 5.00. Comparisons between two values were performed using an unpaired student *t* test. A one-way ANOVA with a *post hoc* Tukey test was used to identify significant differences among groups.

## Conflict of interest

S.D. discloses financial interest in Partikula LLC; Partikula did not support the aforementioned work.

## Supplementary Material

Supplementary information
