# Supplementary material for: Turn up the cellular power generator with vitamin E analogue formulation
Source: Chem Sci. 2016 May 9;7(8):5559–67. doi: 10.1039/c6sc00481d (PMC6022097; doi:10.1039/c6sc00481d)
Supplement: Supplementary file 1 [file SC-007-C6SC00481D-s001.pdf]

Electronic Supplementary Material (ESI) for Chemical Science.
This journal is © The Royal Society of Chemistry 2016

Supporting Information For

Turn Up the Cellular Power Generator with Vitamin E Analogue Formulation

Ru Wen[a] and Shanta Dhar[a]*

[a]NanoTherapeutics Research Laboratory, Department of Chemistry, University of Georgia, Athens, GA 30602

*To whom correspondence should be addressed.

E-mail: shanta@uga.edu; Webpage: http://shanta.uga.edu/

Materials: All chemicals were used without further purification unless otherwise noted. N, N'-dicyclohexylcarbodiimide (DCC), hydrogen peroxide solution (30 wt.% in H_2O), camptothecin, carbonyl cyanide 4-(trifluoromethoxy) phenylhydrazone (FCCP), oligomycin, antimycin A, rotenone, *α*-tocopheryl succinate (*α*-TOS), 3-(4,5-dimethylthiazol-2-yl)-2,5-diphenyltetrazolium bromide (MTT), OH-polyethylene glycol-OH (OH-PEG-OH) of molecular weight 3350, 6-bromohexanoic acid, sodium bicarbonate ($NaHCO_3$), triphenylphosphine, glucose, sodium hydroxide, and tris(hydroxymethyl)aminomethane (Tris) were purchased from Sigma-Aldrich. XF24-well cell culture microplates were purchased from Seahorse Biosciences. 4-dimethylaminopyridine (DMAP) (catalog No. 1122-58-3) was purchased from Alfa Aesar Inc. Poly(DL-lactide-co-glycolide) (PLGA-COOH) with inherent viscosity of 0.15-0.25 dL/g, was purchased from Durect LACTEL® Absorbable Polymers. Bicinchoninic acid (BCA) protein assay kit (Pierce 23227), RIPA buffer (catalog No. 89900), and nitrocellulose membrane (catalog No. 88018) were purchased from Thermo Scientific. Glutamine,

penicillin/streptomycin, trypsin-EDTA solution, HEPES buffer (1 M), and sodium pyruvate were purchased from Sigma Life Sciences. Roswell Park Memorial Institute (RPMI) medium, Dulbecco's Modified Eagle's Medium (DMEM), fetal bovine serum (FBS), 5,5',6,6'-tetrachloro-1,1,3,3-tetraethylbenzimidazolylcarbocyanine iodide (JC-1) dye were purchased from Life Technologies. Annexin V-FITC-propidium iodide (PI) (catalog number 556547) apoptosis detection kit was purchased from BD Biosciences Pharmingen™. Tween 20 was purchased from Fisher Bio-reagent. $CDCl_3$ was purchased from Cambridge Isotope Laboratories Inc. Regenerative cellulose membrane Amicon ultra centrifugal 100 kDa filters (Catalog number UFC910096) were purchased from Merck Millipore Ltd. ATP quantification kit (Catalog number G7570) was purchased from CellTiter-Glo® Promega. Cell-Tak solution for cell and tissue adhesive properties was purchased from Corning® Cell-Tak™ (catalog number. 354240). MitoTox™ Complex V OXPHOS activity microplate assay kit (Catalog number. ab109907), anti-Bcl-2 antibody (Catalog number. ab32124), anti-beta actin antibody (Catalog number. ab8227), and goat anti-rabbit IgG H&L (HRP) preadsorbed (Catalog number. ab97080) were purchased from Abcam Inc. OxiSelect™ Intracellular reactive oxygen species (ROS) assay kit was purchased by Cell Biolabs (Catalog number. STA-342). Amplex® Red hydrogen peroxide/peroxidase assay kit (Catalog number. A22188) was purchased from Thermo Life Technology. Apoptosis inducing factor (AIF) H-300 antibody (Catalog number. sc-5586) was purchased from Santa Cruz Biotechnology. Lactate assay kit (Catalog number. K627-100) was purchased from BioVision Inc. Ammonium persulfate (Catalog number. 161-0180), tris/glycine/SDS buffer (Catalog number. 161-0732), SDS-PAGE gel preparation kit TGX stain-free™ fast cast™ acrylamine 10% (Catalog number. 161-0182),

and Clarity™ western ECL substrate (Catalog number. 170-5060) were purchased from Bio-Rad Inc.

Instruments: Nanopure water was purified by a 0.22 μm filter Millipore Milli-Q Biocel water purification system (18.2 MΩ). Slide-A-Lyzer MINI dialysis tubes were obtained from Thermo Scientific. Proton NMR analyses were carried out on a 400 MHz Varian NMR spectrometer. High-performance liquid chromatography (HPLC) experiments were conducted using an Agilent 1200 LC series instrument equipped with automated injector, and UV and fluorescence detectors. Dynamic light scattering (DLS) measurements were performed on a Malvern Zetasizer Nano ZS system. Transmission electron microscopy (TEM) images were carried out by Philips/FEI Tecnai 20 microscope. Gel permeation chromatographic (GPC) analyses were performed on Shimadzu LC20-AD prominence liquid chromatographer equipped with RI detector. Flow cytometry analyses were performed on Beckman Coulter CyAn™ ADP Analyzer. Cells were counted using a Countess® automated cell counter (Invitrogen Life Technology). Plate reader analyses were performed on a Bio-Tek Synergy HT microplate reader. Mitochondrial bioenergetics assay was performed on a Seahorse XF24 (Agilent Seahorse Biosciences, North Billerica, MA, USA) analyzer. Western blots were imaged on a FluorChem HD2 system from Alpha Innotech (Protein Simple, Santa Clara, CA, USA).

Methods

Cell Line and Cell Culture

Human breast cancer cell line MCF-7, human prostate cancer cell line PC-3, Bcl2 Jurkat, and Neo Jurkat cell line were obtained from the American type culture collection (ATCC). H9C2 cardiomyocytes were received as a generous gift from Prof. Mark Anderson,

University of Iowa. MCF-7, PC-3, Bcl2 Jurkat, and Neo Jurkat cells were grown in RPMI 1640 medium containing 10% FBS, 1% L-glutamine, 1% sodium pyruvate, 1% HEPES, and 1% penicillin/streptomycin at 37 °C in 5% CO_2. H9C2 cell was grown in DMEM with 10% FBS, 1% L-glutamine, 1% sodium pyruvate, 1% HEPES, and 1% penicillin/streptomycin. The cells were passed every 2 or 3 days up to 20 passages and restarted from a new stock.

Synthesis of PLGA-*b*-PEG-OH and PLGA-*b*-PEG-TPP

The PLGA-*b*-PEG-OH and PLGA-*b*-PEG-TPP polymers were prepared by the methods previously reported by our group.[1]

Preparation and Characterizations of *α*-TOS-loaded NPs

The targeted and non-targeted α-TOS nanoparticles (T-*α*-TOS-NPs, NT-*α*-TOS-NPs) were prepared using PLGA-*b*-PEG-TPP and PLGA-*b*-PEG-OH polymers, respectively. A stock solution of the polymer was made by dissolving 5 mg of the polymer in 1 mL of dimethylformamide (DMF). *α*-TOS with different percent feed with respect to polymer was added to the polymer solution. This 1 mL stock solution containing polymer and *α*-TOS was then added drop wise into 10 mL of nanopure water. The mixture was stirred for 2 h and then filtered using Amicon centrifugation filtering device and washed three times using water under centrifugal force of 3000 rpm at 4 °C. Finally the NPs were resuspended in 1 mL water and characterized using DLS and TEM. Size and zeta potential were measured by DLS. The DLS and TEM samples were made by 20 times dilution of the prepared NPs. TEM samples were stained with 4% uranyl acetate. The stained samples (5 *μ*L) were then dropped into the copper grid and air dry for overnight.

The concentration of α-TOS in NPs was quantified by HPLC. A mixture of 50:50 isopropanol: acetonitrile was used as the mobile phase using a wavelength of 292 nm.

Release of *α*-TOS from NPs

To evaluate the release of *α*-TOS from the NPs, the prepared NPs were diluted by 4 times with nanopure water. Then, 100 μL of the diluted solution was added to dialysis tubes. These dialysis tubes were then submerged in phosphate buffered saline (PBS) of pH=7.4 and stirred at 37 °C up to 96 h. The PBS was changed every 4 h for the first 12 h and then changed every 12 h. The samples were collected at predetermined time points and analyzed by HPLC for *α*-TOS concentration.

Cell Viability Evaluation

The cytotoxicity of T-*α*-TOS-NPs and NT-*α*-TOS-NPs in various cell line was studied by the (3-(4,5-dimethylthiazol-2-yl)-2,5-diphenyltetrazolium bromide) (MTT) assay. Specifically, MCF-7 (1500 cells/well), PC-3 (3000 cells/well), H9C2 (2000 cells/well) were seeded in a 96 well plate and grown overnight at 37 °C in 5% CO_2. Then, the media was replenished and NPs at various concentrations were added. For Bcl2 Jurkat and Neo Jurkat cells, 10000 cells/well were seeded in a 96 well plate and the NPs were directly added. After 12 h, the media was removed and fresh media was added. After another 60 h of further incubation, MTT reagent (5 mg/mL, 20 μL/well) was added. Upon 5 h incubation, MTT containing media was removed and cells were lysed using 100 μL of DMSO, followed by 5 min gentle shaking at room temperature. The sample absorbance was monitored at 550 nm using a background absorbance at 800 nm. Cytotoxicity was expressed as mean percentage ± standard deviation (S.D.) relative to the untreated control. Cytotoxicity data was fitted by a sigmoidal curve and IC_{50}, the concentration

which inhibits 50% cell growth compared to untreated control, was calculated by a four parameters logistic model. All these analyses were performed with GraphPad Prism (San Diego, U.S.A).

Evaluation of Mitochondrial Membrane Potential

Bcl2 Jurkat and Neo Jurkat cells (1×10^6 cells /well) were plated in 12-well plates. NPs with concentration range of 20-40 μM with respect to α-TOS was added and incubated for 12 h. As positive control, FCCP at a concentration of 10 μM was added and incubated for 30 min. 5,5′,6,6′-tetrachloro-1,1′,3,3′-tetraethylbenzimidazolylcarbocyanine iodide or JC-1 was added to each well at a final concentration of 2 μg/mL and incubated for 30 min at 37 °C. The stained samples were harvested by centrifugation at 1500 rpm for 5 min (4 °C) and washed with 1 mL PBS for two times. The cells were then resuspended in 100 μL PBS and fluorescence was read at both 485/528 and 530/590 nm.

Apoptosis Assay

Bcl2 Jurkat and Neo Jurkat cells ($1\text{x}10^6$ cells/well) were plated in 12-well plate and 25 μM NPs with respect to α-TOS or free α-TOS at the same concentration were added and incubated for 12 h. A positive and negative control for early apoptosis and necrosis were also used by adding camptothecin (20 μM for 12h) and H_2O_2 (100 mM for 45 min), respectively. The cell pellets were collected by centrifuge with 1500 rpm for 5 min at 4 °C. After 3 washes with cold PBS, the cells were resuspend in 100 μL 1X annexin-binding buffer stained with Annexin V-FITC and propidium iodide for 15 min at room temperature, then 400 μL 1X annexin-binding buffer was added, and the samples were analyzed by flow cytometry.

Bicinchoninic acid (BCA) assay

The protein quantification was carried out by BCA assay. Albumin was used as standard. Samples were incubated with 200 *μ*L of working solution composed of reagent A: reagent B=50:1 at 37º C for 30 min, and then the absorbance was measured at 562 nm.

Immune Blotting

Bcl2 Jurkat and Neo Jurkat cells ($1×10^6$ cells/well) were plated into the 24-well plate and treated with *α*-TOS, NT-*α*-TOS-NPs, or T-*α*-TOS-NPs (20 *μ*M with respect to *α*-TOS) for 12 h. The cells were harvested by centrifugation at 1500 rpm for 5 min at 4 °C and washed with PBS twice. Radioimmunoprecipitation assay (RIPA) buffer was added into the cells and then incubated on ice for 30 min. The supernatants were collected as the samples after centrifugation at 12000 *g* for 10 min at 4 °C. The protein concentrations in the samples were quantified by the BCA assay. Proteins were resolved in a SDS-PAGE gel, transferred onto a nitrocellulose membrane, and incubated with primary antibody (Bcl2, AIF, *β*-actin) at 4 °C overnight, and then incubated with secondary antibodies at room temperature for 2 h. The membranes were developed with enhanced chemiluminescence (ECL) for imaging. The bands in membranes were quantified by Image J.

ATP Synthase Activity Assay

The mitochondria ATP synthase activity was assessed by the complex V OXPHOS activity assay kit from Abcam. ATP synthase was exposed to reagents by solubilizing the bovine heart mitochondria (BHM). 40 *μ*L of detergent was mixed with 360 *μ*L BHM (5.5 mg/mL) and incubated on ice for 30 min to solubilize the mitochondria. The supernatant solubilized BHM was collected by centrifugation at 12,000xg for 20 min at 4 °C and

diluted with 5 mL of 1X Mito-Buffer. The exposed ATP synthase with respect to 0.5 μg/mL protein was treated with either 30 μM of NPs with respect to α-TOS in 200 μL of complex V activity buffer containing ATP, pyruvate kinase (PK), lactate dehydrogenase (LDH), phosphoenolpyruvate, NADH. As controls, oligomycin (200 nM)-treated and non-treated samples were analyzed. The absorbance was measured at 340 nm.

MitoStresss Assay

Bcl2 Jurkat (1×10^6 cells/well) were plated into the 24-well plate and treated with α-TOS, NT-α-TOS-NPs, or T-α-TOS-NPs (20 μM with respect to α-TOS) for 12 h. The XF cartridge was hydrated with 1 mL calibrant for 12 h in a CO_2 free environment at 37 °C incubator. The coated XF microplate was prepared by 1 h incubation at 37 °C with 50 μL coating solution containing 22.4 μg/mL of Cell-Tak solution in neutral buffer. A volume of 1 M NaOH equal to half the volume of Cell-Tak solution was used to bring the solution to neutral. Then the microplate was washed with tissue culture grade sterile water 3 times and air-dried for 10 min. The cells were collected via centrifugation at 1500 rpm for 5 min at 4 °C and washed with cold PBS twice. Assay medium was prepared by DMEM (pH=7.42) containing 1% L-glutamine, 1% sodium pyruvate, and 1% glucose. After resuspension in 0.5 mL assay medium, the cells (100 μL) were seeded into the coated XF microplate and centrifuged to have the cells adhered to the bottom. The centrifuge speed range was adjusted to low for DEC (deceleration). Once the centrifuge speed reached to 450 rpm, it was stopped. The coated XF microplate was then rotated 180° and centrifugation stopped once the speed reached 650 rpm. After the cells were incubated in a CO_2 free environment at 37 °C for 20 min, 450 μL of assay medium was added, and incubated for another 30 min. Finally, the samples were analyzed by MitoStress assay

(Agilent Seahorse Bioscience) with injection of oligomycin (1.0 μM), FCCP (1.0 μM), antimycin-A (1.0 μM), and rotenone (1.0 μM) using A, B, and C ports in hydrated cartridge, respectively.

For H9C2 cells, 20,000 cells/well were plated in a 24-well microplate and grown for overnight. The medium was replenished and cells were treated with α-TOS, NT-α-TOS-NPs, or T-α-TOS-NPs (20 μM with respect to α-TOS) for 12 h. The medium was removed by taking 150 μL out and washed 3 times with adding and removing of 450 μL of assay medium. Finally, 450 μL of assay medium was added and incubated for 1 h. Finally, the samples were analyzed by MitoStress assay as described before for Bcl2 Jurkat cells.

CellTiter-Glo® Luminescent ATP Quantification

ATP quantification was carried out by CellTiter-Glo® Cell Viability Assay kit. MCF-7 and H9C2 cells (50,000 cells/well) were plated in 96-well plates in 100 μL medium and grown for overnight. Then the medium was replenished and cells were treated with α-TOS, NT-α-TOS-NPs, or T-α-TOS-NPs (40 μM with respect to α-TOS) for 5 h at 37 °C in 5% CO_2 atmosphere. As a control, oligomycin (100 μM for MCF-7, 25 μM for H9C2) treatment was performed. The plates were equilibrated at room temperature for 30 min. 100 μL reagent was added into the 96 well plate and mix for 2 min to ensure the cell lysis. The luminescence was recorded with 10 min delay at room temperature.

ROS Assay

Intracellular ROS was assessed by the ROS assay Kit from Cell Biolabs. MCF-7 and H9C2 cells (50,000 cells/well) were plated in 96-well plates in 100 μL medium and grown for overnight. Then the medium was replenished and cells were treated with α-TOS, NT-α-TOS-NPs, or T-α-TOS-NPs (40 μM for MCF-7, 60 μM for H9C2 with respect to α-TOS)

for 12 h at 37 °C in 5% CO_2 atmosphere. As a control, oligomycin (25 μM) treatment was performed. The media was removed and 100 μL of 1X dichloro-dihydro-fluorescein diacetate (DCFH-DA)/media solution was added to the cells and incubated for 45 min. The solution was removed and washed with PBS twice. Finally, RIPA cell lysis buffer was added and mixed for 2 min. The fluorescence was recorded at 485/530 nm.

Cellular H_2O_2 Production Assay

H_2O_2 production by cells was quantified by Amplex Red-hydrogen peroxide/peroxidase assay kit. MCF-7 (50,000 cells/well) were plated in 96-well plates and grown for overnight. The medium was replenished and the cells were treated with malonate (100 mM), *α*-TOS, NT-*α*-TOS-NPs, or T-*α*-TOS-NPs (40 μM with respect to *α*-TOS) with or without malonate for 24 h at 37 °C. To each well, 100 μL Amplex Red working substrate containing 100 μM Amplex® Red reagent and 0.2 U/mL horseradish peroxidase was added and incubated for 30 min. A standard curve of H_2O_2 was constructed in medium for quantification. The fluorescence was measured at 530/590 nm.

Cellular Lactate Analyses

Cellular lactate level was analyzed using a Biovision lactate analysis kit. MCF-7 cells (50,000 cells per well) were plated in a 96-well plate and grown for overnight. The medium was replenished and the cells were treated with *α*-TOS, NT-*α*-TOS-NPs, or T-*α*-TOS-NPs at a concentration of 40 μM with respect to *α*-TOS. Oligomycin (25 μM) was used as a control. Incubation was carried out for 5 h. The medium was removed and 50 μL of RIPA buffer was used to lyse the cells, followed by addition of 50 μL of reaction mix containing 46 μL lactate assay buffer, 2 μL lactate probe, and 2 μL lactate enzyme mix.

The absorbance was measured at 450 nm after incubation for 30 min at room temperature in the dark.

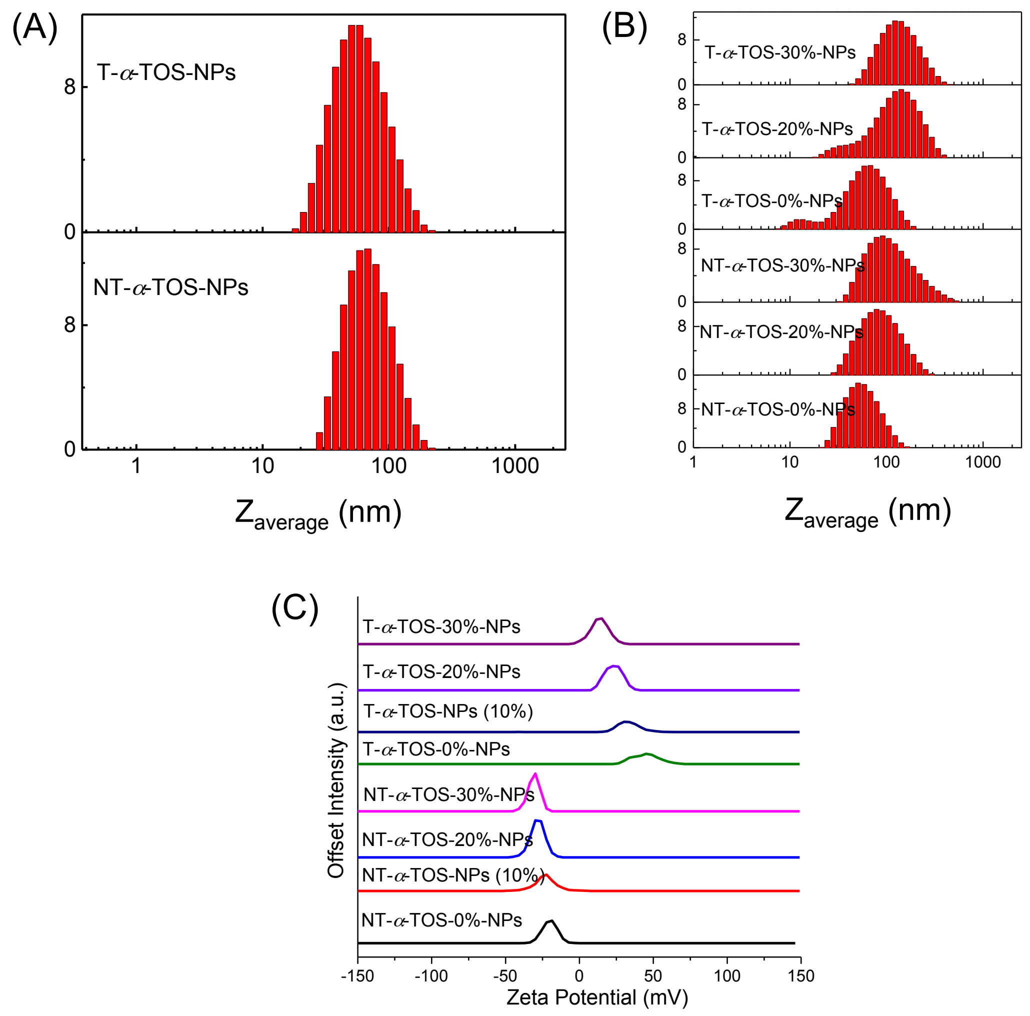

Figure S1. (A) Hydrodynamic diameter ($Z_{average}$) of T-*α*-TOS-NPs and NT-*α*-TOS-NPs prepared with 10% feed of *α*-TOS. (B) Hydrodynamic diameter ($Z_{average}$) of T-*α*-TOS-NPs and NT-*α*-TOS-NPs prepared with varied feed of *α*-TOS demonstrating that the NPs with 10% feed have monodisperse population. (C) Zeta potential of T-*α*-TOS-NPs and NT-*α*-TOS-NPs prepared with varied feed of *α*-TOS. T-*α*-TOS-NPs demonstrated decreased positive surface charge as the percent *α*-TOS increased and NT-*α*-TOS-NPs also showed less negatively charged surface as the *α*-TOS feed was increased.

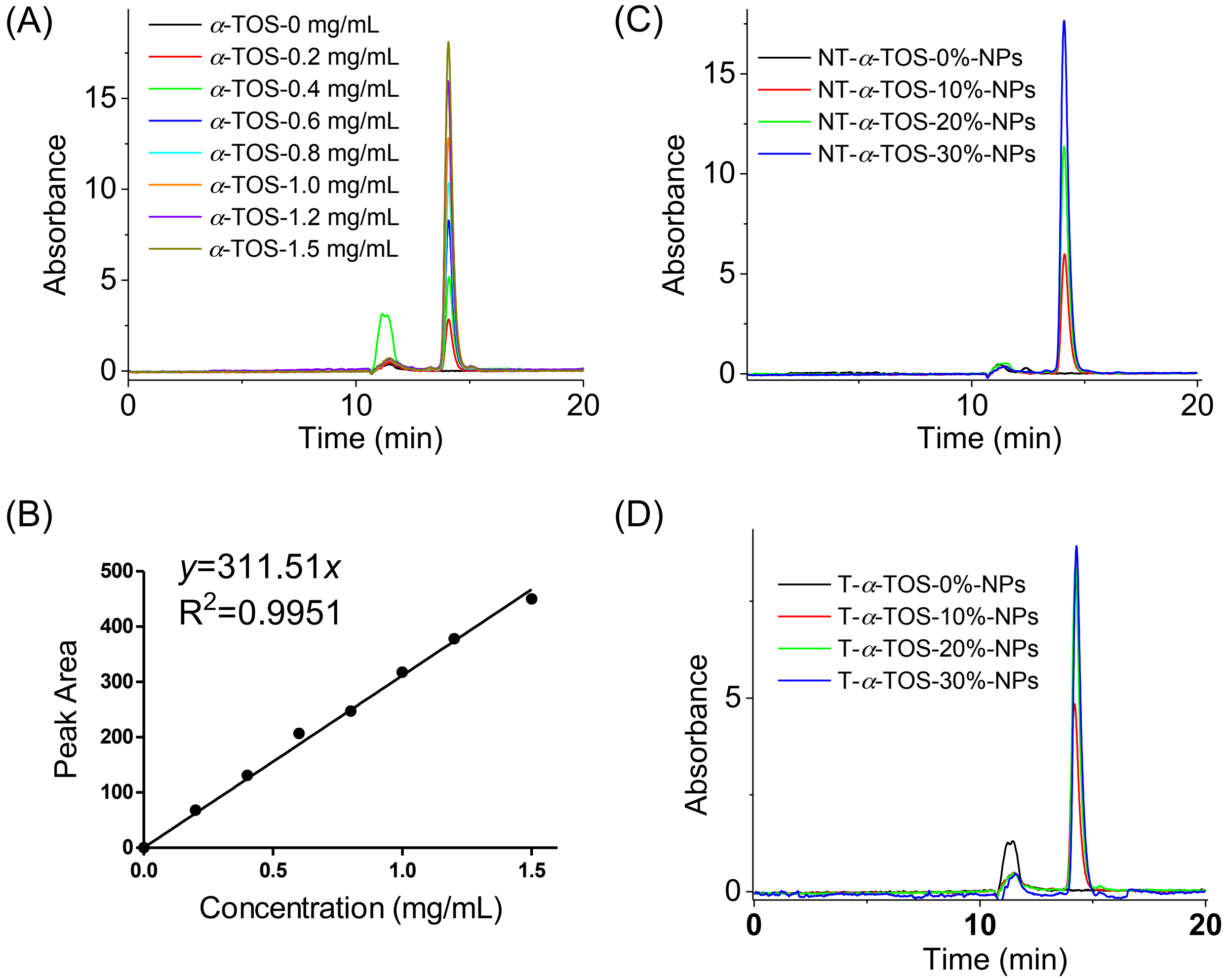

Figure S2. (A) Representative high performance liquid chromatography (HPLC) chromatograms of *α*-TOS at various concentrations as standard and (B) corresponding calibration curve. Representative HPLC chromatograms of (C) NT-*α*-TOS-NPs and (D) T-*α*-TOS-NPs. The peak area with retention time centered at ~14 min was plotted in the calibration curve and the concentration of T/NT-*α*-TOS-NPs was calculated by using the calibration curve.

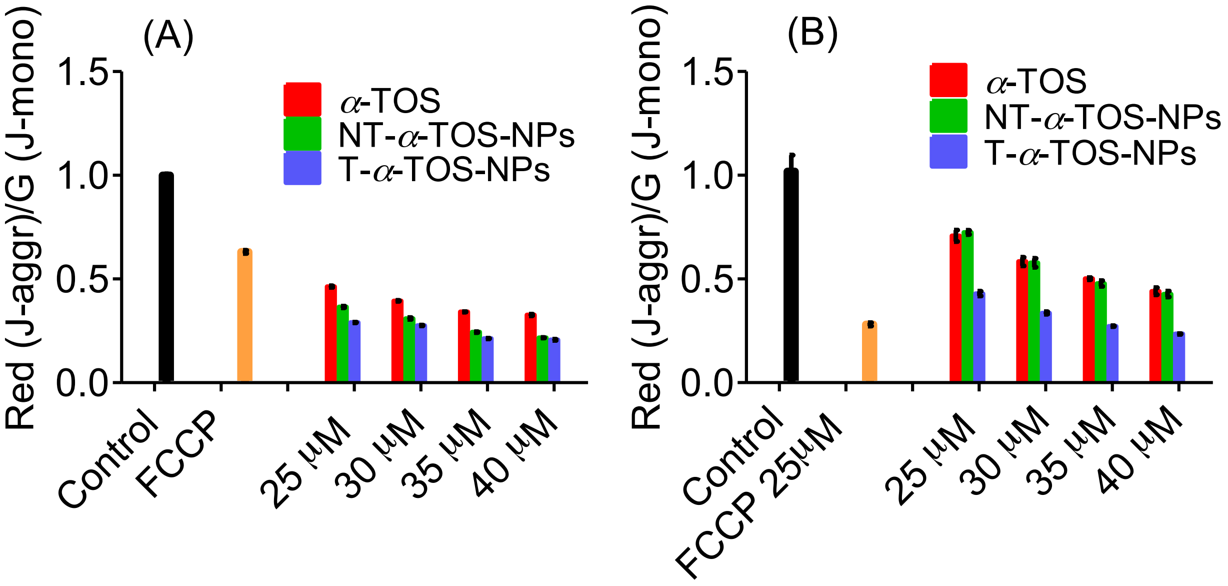

Figure S3. Representative JC-1 aggregate to monomer ratio in (A) Bcl2 Jurkat cells and (B) Neo Jurkat cells after treatment with *α*-TOS, T/NT-*α*-TOS-NPs at varied concentrations with respect to *α*-TOS.

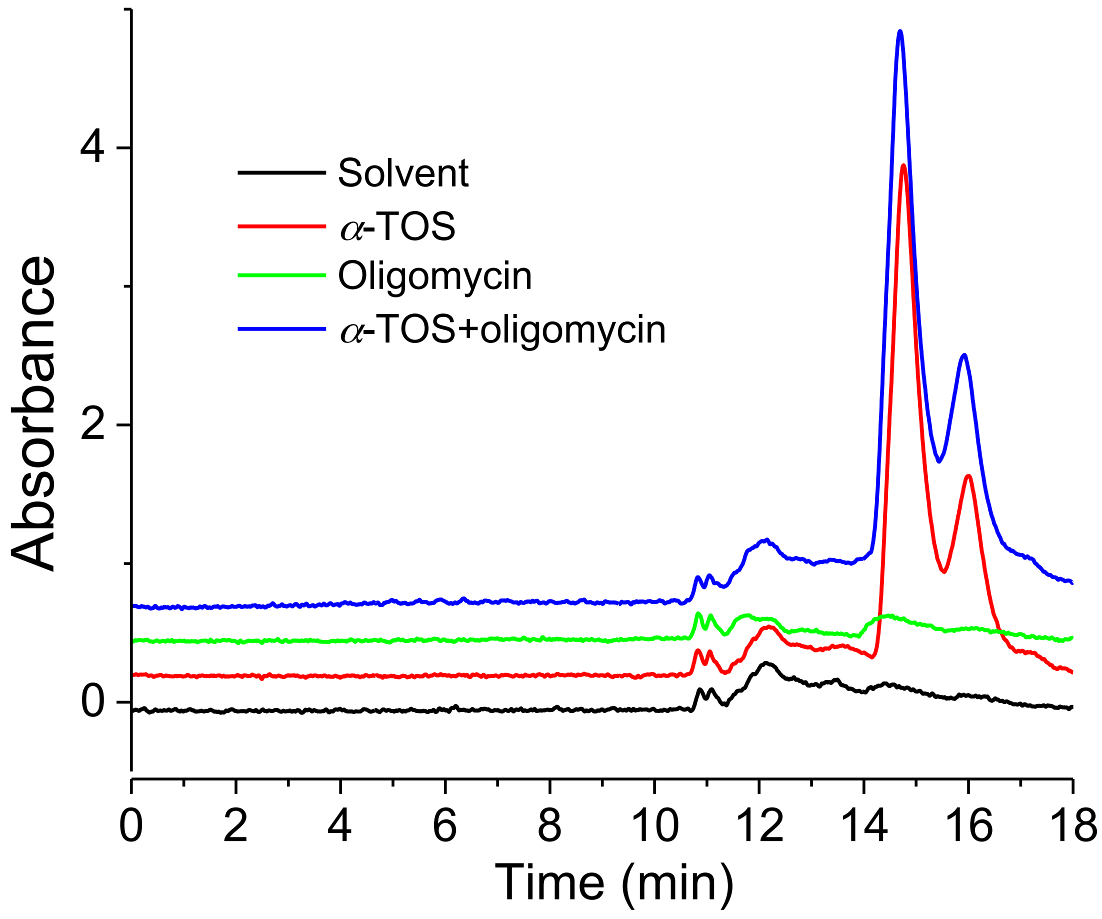

Figure S4. Representative HPLC chromatograms of *α*-TOS, oligomycin, *α*-TOS and oligomycin combination ruling out any possibility of complex formation between α-TOS and oligomycin.

References:

1. (a) S. Marrache and S. Dhar, *Proc. Natl. Acad. Sci. U.S.A.*, 2012, **109**, 16288-16293; (b) S. Marrache, R. K. Pathak and S. Dhar, *Proc. Natl. Acad. Sci. U.S.A.*, 2014, **111**, 10444-10449; (c) S. Marrache, S. Tundup, D. A. Harn and S. Dhar, *ACS Nano*, 2013, **7**, 7392-7402.
